# Farnesoid X receptor alpha ligands inhibit HDV *in vitro* replication and virion infectivity

**DOI:** 10.1097/HC9.0000000000000078

**Published:** 2023-04-14

**Authors:** Anne-Flore Legrand, Julie Lucifora, Benoît Lacombe, Camille Ménard, Maud Michelet, Adrien Foca, Pauline Abrial, Anna Salvetti, Michel Rivoire, Vincent Lotteau, David Durantel, Patrice André, Christophe Ramière

**Affiliations:** 1 CIRI, Centre International de Recherche en Infectiologie, Team VIRIMI, Univ Lyon, Inserm, Université Claude Bernard Lyon 1, CNRS, ENS de Lyon, Lyon, France; 2 University of Lyon, Université Claude Bernard Lyon, Villeurbanne, France; 3 CIRI, Centre ge International de Recherche en Infectiologie, Team HepVir, Univ Lyon, Inserm Université Claude Bernard Lyon 1, CNRS, ENS de Lyon, Lyon, France; 4 INSERM U1052, Cancer Research Center of Lyon (CRCL), University of Lyon (UCBL1), CNRS Centre Léon Bérard, Lyon, France; 5 INSERM Centre Léon Bérard (CLB), Lyon, France; 6 Virology Laboratory, Hospices Civils de Lyon, Hôpital de la Croix-Rousse, Lyon, France

## Abstract

HDV, a satellite of HBV, is responsible for the most severe form of human viral hepatitis, for which curative therapy is still awaited. Both HBV and HDV use the hepatic transporter of bile acids (ie, Na+-taurocholate cotransporting polypeptide) to enter hepatocytes. We have previously shown that ligands of the farnesoid-X-receptor alpha (FXR), a master regulator of bile acids metabolism, inhibit HBV replication. Here we asked whether FXR ligands can also control HDV infection.

**Approach and Results:**

*In vitro* HDV monoinfections or HDV/HBV coinfections and superinfections were performed in differentiated HepaRG cells (dHepaRG) and primary human hepatocytes. Following treatment with FXR ligands, HDV RNAs and antigens were analyzed by RT-qPCR, northern blot, immunofluorescence, and western blot. Virus secretion was studied by RNA quantification in supernatants, and the infectivity of secreted HDV particles was measured by reinfection of naive HuH7.5-Na+-taurocholate cotransporting polypeptide cells. In HDV/HBV superinfection models, a 10-day treatment with FXR ligand GW4064 decreased intracellular HDV RNAs by 60% and 40% in dHepaRG cells and primary human hepatocytes, respectively. Both HDV genomic and antigenomic RNAs were affected by treatment, which also reduced the amount of intracellular delta antigen. This antiviral effect was also observed in HDV monoinfected dHepaRG cells, abolished by FXR loss of function, and reproduced with other FXR ligands. In HBV/HDV coinfected dHepaRG cells, HDV secretion was decreased by 60% and virion-specific infectivity by >95%.

**Conclusions:**

FXR ligands both inhibit directly (ie, independently of anti-HBV activity) and indirectly (ie, dependently of anti-HBV activity) the replication, secretion, and infectivity of HDV. The overall anti-HDV activity was superior to that obtained with interferon-α, highlighting the therapeutic potential of FXR ligands in HDV-infected patients.

## INTRODUCTION

HDV is a defective and satellite virus of HBV, requiring HBsAg for its propagation. HDV coinfection or superinfection in HBV carriers is characterized by a more rapid progression to liver fibrosis and an increased risk of end-stage and lethal liver diseases.^1^,^2^ The exact prevalence of HDV infection is unknown. According to recent meta-analyses, it is estimated that HDV infects between 12 and 60 million people worldwide, which corresponds to 4.5%–13% of HBV-infected patients, with significant geographic variations of prevalence.^1^,^3^,^4^

Current therapies relying mainly on pegylated interferon-alpha (Peg-IFN-α) are unsatisfactory, as a sustained virologic response (SVR) following cessation of treatment is only obtained in a very limited number of patients.^5^,^6^ Recently, the HDV entry inhibitor Bulevirtide (also widely known as Myrcludex) was approved in the European Union for the treatment of HDV patients with compensated liver disease, but optimal treatment duration and SVR remain to be determined.^7^ A few investigational antiviral approaches are currently being tested, but it is widely admitted that novel specific antiviral strategies will be necessary to foster an HDV cure.

Replication of the HDV genome, a 1.7 kb single-stranded negative-sense circular RNA, takes place in the nucleus of infected hepatocytes. Antigenomic RNAs are synthesized by a rolling circle mechanism and serve as templates for the synthesis of new genomic RNAs. Viral mRNAs are also synthesized and encode the small and large delta antigens (HDAg-S and HDAg-L) from a unique open reading frame.^8^ Compared with HDAg-S (195 amino-acid long), HDAg-L contains an additional domain of 19–20 AA at its C-terminus, which results from an adenosine deaminase RNA-specific 1–mediated RNA editing of the antigenomic HDV RNA at a location corresponding to the stop codon of the HDAg-S gene.^9^ This additional domain contains a CXXX-box motif, allowing the addition of a farnesyl group to the cysteine by cellular farnesyltransferase activity. While HDAg-S is essential to HDV replication, farnesylated HDAg-L inhibits the replication step but favors virus egress.^10^,^11^ HDV assembly is initiated by the interaction between HDAg-L and newly synthesized genomic RNAs. These neo-formed ribonucleoproteins, which also contain HDAg-S moieties,^12^ are then exported from the nucleus to bud into HBsAg empty subviral particles (ie, devoid of HBV nucleocapsids), supposedly at the endoplasmic reticulum, to generate HDV infectious particles.^13^ Aside from the crucial role of HBV envelope proteins in the production of infectious HDV particles, the other steps of the HDV life cycle are not dependent on HBV.

Bearing HBV envelope proteins at their surface, HDV infectious virions use the same entry receptor as HBV to enter hepatocytes, that is the sodium taurocholate cotransporting polypeptide (NTCP).^14^ NTCP is the main transporter of bile acids (BA) at the basolateral membrane of hepatocytes and several studies have suggested that HBsAg-containing particles and BAs compete for binding to NTCP. Indeed, it has been shown that high concentrations of taurocholate inhibit both HBV and HDV entry into differentiated HepaRG cells (dHepaRG). Inversely, binding of the HBsAg preS1 domain to NTCP blocks NTCP-mediated BA uptake in HepG2-NTCP cells.^15^,^16^ Even though HDV uses NTCP to enter hepatocytes, the link between BA metabolism and HDV life cycle remains to be explored. BA metabolism homeostasis is maintained by a complex interplay between the liver and the gut, in which the farnesoid-X-receptor alpha (FXR), the liver-enriched nuclear receptor (NR) of BA, plays a key role by directly or indirectly regulating the transcription of numerous genes. FXR activation in response to increased intracellular BA concentrations leads to its downregulation (through a negative feedback loop), a decreased BA entry into enterocytes and hepatocytes, as well as an increased BA excretion into bile ducts through the regulation of expression of several BA transporters (eg, the bile salt export pump, BSEP). In addition, synthesis of primary BA from cholesterol is inhibited in the liver, in particular following repression of cytochrome P450 Family 7 Subfamily A Member 1 (CYP7A1) enzyme expression.^17^

In our previous work, we have shown that FXR can bind to 2 FXR response elements on the HBV genome.^18^ Other studies have shown that HBV infection modified the expression of FXR and its target genes in the humanized mouse model and liver biopsies from chronically infected patients.^19^ Importantly, we and others have shown that some FXR agonists inhibit HBV replication *in vitro*.^20^,^21^ This led to the clinical evaluation, in monotherapy or combination with standards of care (SoC), of the FXR agonist Vonafexor, as a potential anti-HBV asset.^22^

Here, we address the question of whether FXR and BA metabolism could also play a role in the HDV life cycle, and whether this could be of therapeutic interest. Using relevant cell culture models [primary human hepatocytes (PHH) and dHepaRG cells], we showed that treatment with FXR ligands leads to a moderate inhibition of HDV intracellular replication; irrespective of the HBV presence, there is a strong decrease of HDV virion secretion, and a very potent reduction of their specific infectivity. These data open perspectives for the treatment of hepatitis delta with FXR ligands.

## METHODS

### Plasmids and viruses

HDV-1 replication-competent plasmid (pSVLD3) and HBV envelope protein (genotype D) encoding the pT7HB2.7 plasmid were kind gifts from Camille Sureau (INTS). HDV viral stocks were obtained by transfection of HuH7 cells with plasmids pSVLD3 and pT7HB2.7, as previously described.^23^ HBV viral stocks were produced from HepAD38 cells as described before.^24^

### Chemicals

FXR agonist GW4064 was purchased from Sigma-Aldrich, 6α-ethylchenodeoxycholic acid (6-ECDCA) from MedChemExpress, and tropifexor from ProbeChem.

Interferon-alpha (IFN-α)-2a was purchased from PBL Assay Science and lamivudine from Selleckchem.

### Cell culture and infections

HepaRG cells were cultured, differentiated, and infected by HBV and HDV as described.^25^,^26^

PHH were freshly prepared from a human liver resection obtained from the Centre Léon Bérard (Lyon) with French ministerial authorizations (AC 2013-1871, DC 2013–1870, AFNOR NF 96 900 September 2011) as described.^27^

A polyclonal HepaRG-TR-Cas9 cell line was generated by dual lentiviral transduction (T-Rex system from Invitrogen/Thermo Fischer) leading to the stable chromosomic integration of 2 transgenic expression cassettes, one constitutively coding the tetracycline repressor protein (TR) and another coding upon tetracycline induction (2 binding sequences for TR in the promoter/+1 transcription region) the *Streptococcus pyogene* Cas9 protein.

HuH7.5 cells were kindly provided by C.M. Rice (Rockefeller University). The derived HuH7.5-NTCP cells were generated by lentiviral transduction.^28^

### Analysis of specific infectivity and density of secreted HDV particles

Supernatants from dHepaRG infected with both HBV and HDV were concentrated using 8% PEG 8000. HDV RNA was quantified by RT-qPCR in concentrates and HuH7.5-NTCP cells were infected using the same viral genome equivalents for each condition of treatment. At indicated times, total cellular RNA was extracted, and HDV RNA was quantified by RT-qPCR.

In parallel, concentrated viruses were characterized by analysis of fractions from 20% to 44% iodixanol gradients. Twelve fractions of 1 mL were collected and used for the quantification of HDV RNA and HBV DNA by qPCR, HBsAg dosage by ELISA, and HDAg and HBsAg detection by western blot.

Methods for nucleic acid quantification, small interfering RNA transfection, HBs and HBe quantification by ELISA, and western blot and immunofluorescence analyses are described in the Supplemental Methods section (http://links.lww.com/HC9/A175). Antibodies used for western blot and immunofluorescence experiments are listed in supplementary table 1.

## RESULTS

### FXR ligand GW4064 decreases intracellular levels of HDV RNAs and proteins in both HBV-infected dHepaRG cells and PHH superinfected with HDV

**FIGURE 1:**
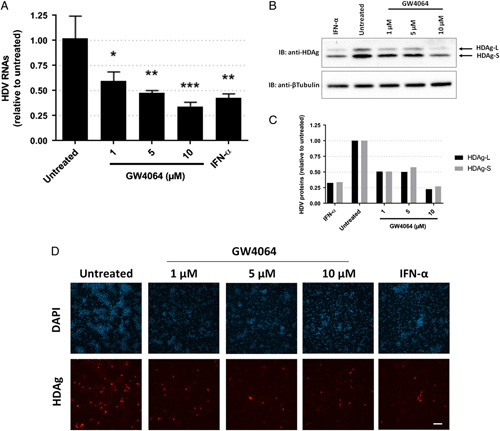
Farnesoid X receptor alpha ligand GW4064 decreases the levels of intracellular HDV RNAs and proteins in HBV-infected differentiated HepaRG superinfected with HDV. Differentiated HepaRG cells were infected with HBV at an MOI of 100 viral genome equivalent (vge) per cell and 7 days later with HDV at an MOI of 10 vge per cell. Three days after HDV infection, cells were treated with 1, 5, or 10 µM of GW4064, IFN-α (1000 IU/mL), or not. Cells were harvested 10 days after treatment for cellular RNA and protein extraction or fixed with formaldehyde for immunofluorescence analyses. Intracellular HDV RNAs were quantified (A). Results are the mean ±SD of 3 experiments each performed with 3 biological replicates. Student *t* test *p<0.05, **p<0.01, ***p<0.001. Analysis of the levels of HDAg by western blot analyses was performed using anti-HDAg antibodies and anti-B-tubulin antibodies as a loading control (B). Densitometry analyses are presented as ratios of HDAgs normalized to the levels of B-tubulin (C). Immunofluorescence analyses were performed using anti-HDAg antibodies and nuclei DAPI staining (D). Scale bar: 200 µm.

The anti-HDV activity of the synthetic FXR ligand GW4064 was first evaluated in dHepaRG cells infected with HBV and superinfected with HDV; such a protocol was initially used as it best mimics *in vivo* infections. IFN-α, which can be considered as the current SoC for HDV in its pegylated form, was used as a control. In superinfected dHepaRG cells, a 10-day treatment with GW4064 decreased the amount of total intracellular HDV RNAs in a dose-dependent manner, reaching 66% of inhibition at 10 µM of GW4064 (Figure 1A). Of note, the same levels of inhibition were obtained with a supraphysiological dose of IFN-α (1000 IU/mL), thus suggesting a superiority of the FXR agonization over type-I IFN receptor engagement.

Then, we analyzed intracellular HDV antigens, that is HDAg-S and HDAg-L by western blot and immunofluorescent staining. Treatment with GW4064 decreased intracellular amounts of both HDAg-S and HDAg-L in the same proportions, by 50% at 1 and 5 µM of GW4064 and by 75% for 10 µM (Figure 1B, C). Once again, 10 µM of GW4064 led to the same inhibition as with IFN-α. Moreover, we observed less HDAg-positive cells following treatments (Figure 1D).

The effect of GW4064 on HDV was also analyzed in PHH infected with HBV and superinfected with HDV. Following treatment with FXR ligand GW4064, a 44% reduction of intracellular HDV RNA levels was observed at 10 µM (Figure S1A, http://links.lww.com/HC9/A176). Western blot and IF analyses also showed a decrease of HDV antigens in infected PHH (Figure S1B–D, http://links.lww.com/HC9/A176). The anti-HDV activity of GW4064 was slightly lower in PHH than in dHepaRG cells, most likely reflecting a much higher replication of HDV in PHH.^28^

HBV infection was also monitored by measuring total intracellular HBV RNAs and HBeAg levels secreted in cell culture supernatants. As expected based on previous studies,^20^ GW4064 strongly inhibited both markers of HBV replication in either superinfected dHepaRG cells or PHH (Figure S2, http://links.lww.com/HC9/A176).

Overall, these results showed an anti-HDV activity of the FXR ligand GW4064 both at the RNA and protein level in two relevant *in vitro* models of HBV/HDV superinfection.

### FXR ligands inhibit early phases of HDV infection in dHepaRG cells and PHH monoinfected with HDV

**FIGURE 2:**
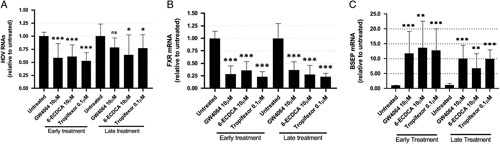
Farnesoid X receptor alpha (FXR) ligands inhibit early and late phases of HDV infection in HDV-monoinfected dHepaRG cells. Differentiated HepaRG cells were infected with HDV at an MOI of 10 viral genome equivalent per cell. Cells were treated with 10 µM of GW4064 or 6-ECDCA or 0.1 µM of tropifexor, 1 day postinfection for early treatment, and 5 days postinfection for late treatments. Cells were harvested 10 days after treatment. Total cellular RNAs were extracted and intracellular HDV RNAs (A), FXR mRNA (B), and BSEP mRNA (C) were quantified by RT-qPCR. Results are the mean±SD of 3 experiments each performed with 3 biological replicates. Data are normalized to untreated conditions for early and late treatments. Student *t* test, *p<0.05, **p<0.01, ***p<0.001. Abbreviations: BSEP, bile salt export pump; ns, not significant.

To further describe the inhibitory effect of FXR ligands on HDV infection, we analyzed the impact of FXR ligands whether treatment was initiated at early times postinfection or after the peak of HDV replication, to determine whether FXR modulation affected infection establishment or later phases of the HDV life cycle. To this end, HDV monoinfection experiments were performed in dHepaRG cells, thus freeing a potential effect of FXR ligands on HBV infection. Following infections, cells were treated at day 1 postinfection (early treatment) or at day 5 postinfection (late treatment). Moreover, to rule out putative off-target effects of GW4064, the cells were treated for 10 days with 3 structurally different FXR ligands, a BA derivate (6-ECDCA) and 2 nonsteroidal synthetic ligands (GW4064 and tropifexor). Quantification by RT-qPCR at the end of treatment showed that GW4064, 6-ECDCA, and tropifexor reduced the amount of total intracellular HDV RNAs by around 50% following early treatment (Figure 2A). A decrease in HDV RNAs was also observed after late treatment. However, this decrease was overall less pronounced and not significant with GW4064. For both early and late treatments, FXR ligands significantly decreased the level of FXR mRNA and strongly increased the level of BSEP mRNA as expected^29^ (Figure 2B, C). As the different forms of viral RNAs produced in HDV-infected cells could not be discriminated by our RT-qPCR, we verified by northern blot analysis the effect of FXR ligands on genomic and antigenomic RNAs. Results showed that the levels of both genomic and antigenomic RNAs were decreased following treatment with all 3 FXR ligands (Figure S3, http://links.lww.com/HC9/A176). Similar experiments of early and late treatments were performed in PHH. Contrary to dHepaRG cells, an antiviral effect of FXR ligands was only observed when treatment was initiated at early phases of HDV infection. The maximal inhibition was obtained with tropifexor with a 60% decrease of HDV RNAs compared with a 30% decrease with GW4064. When infection was established, FXR ligands were not efficient to impede HDV replication (Figure S4, http://links.lww.com/HC9/A176). As PHH maintain detoxification capacities, these differences may result from different kinetics of FXR ligands catabolism in these cells. Importantly, treatment did not significantly modify hepatocyte nuclear factor-4 mRNA expression compared with untreated cells, suggesting that the evolution of the differentiation state of PHH was not strongly affected by FXR ligands (Figure S5, http://links.lww.com/HC9/A176). Together, these results showed that 3 FXR agonists displayed antiviral activity against HDV, especially on early HDV infection phases in 2 relevant *in vitro* models. An antiviral effect of a late treatment was only observed in dHepaRG cells. The use of 3 structurally different FXR ligands showed that the anti-HDV activity of GW4064 was not the result of an off-target effect. Moreover, the inhibitory effect of FXR ligands did not require the presence of HBV.

### Antiviral activity of FXR ligands is specific and relies on the presence of FXR

**FIGURE 3:**
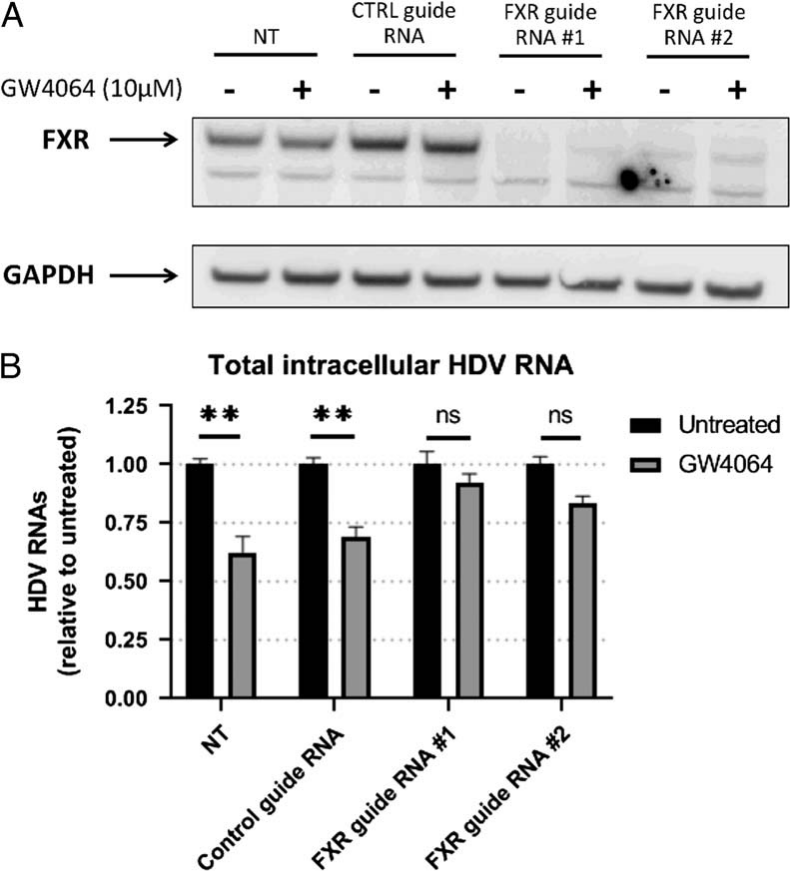
FXR ligands decrease the levels of intracellular HDV RNAs in an FXR-dependent manner. Differentiated HepaRG-TR-Cas9 cells were transfected twice with indicated RNA guides 4 days and 1 day before infection with HDV at an MOI of 25 viral genome equivalent per cell. From day 3 to day 9 postinfection, cells were treated with 10 µM of GW4064 or vehicle. Cells were harvested at day 9 postinfection. (A) Levels of FXR proteins were analyzed by the western blot. GAPDH detection was used as a loading control. (B) Total cellular RNAs were extracted and the levels of total intracellular HDV RNAs were quantified by RT-qPCR. Results are the mean±SD of 3 experiments each performed with 3 biological replicates. Data are normalized to the untreated cells for each condition of transfection. Two-way ANOVA, **p<0.01. Abbreviations: FXR, Farnesoid X receptor alpha; ns, not significant; NT, nontransfected.

FXR ligands used in these experiments, in particular GW4064 and tropifexor, are considered highly specific for FXR. However, to confirm that the antiviral activity of these molecules was indeed FXR-specific, we silenced FXR in HDV-monoinfected cells. We took advantage of an HepaRG cell line expressing an inducible Cas9 (described in Figure S6, http://links.lww.com/HC9/A176) to use the CRISPR-Cas9 technology to stably repress FXR expression. Two synthetic crRNA targeting distinct exons of the FXR gene were used. Transfection of either of the 2 guide RNAs led to a strong decrease in FXR protein level, indicating that both guides were efficient to block FXR protein synthesis (Figure 3A). In line with our previous results, a 6-day treatment with GW4064 moderately, but significantly, reduced the amount of total intracellular HDV RNAs in nontransfected cells and in cells transfected with a control crRNA (Figure 3B). In FXR-silenced cells, the inhibitory effect of GW4064 was either completely (case of guide #1) or greatly (case of guide #2) reverted, confirming that its anti-HDV activity was indeed dependent on FXR expression. To confirm these results, we also silenced FXR in dHepaRG cells coinfected with HBV and HDV by using small interfering RNA technology. The antiviral effect of 3 FXR ligands was reverted in the presence of small interfering RNA targeting FXR, whose expression was decreased by more than 80% (Figure S7A, B, http://links.lww.com/HC9/A176). The induction of BSEP expression was also reduced in the presence of siFXR after FXR ligand treatment compared with the siCTRL untreated condition (Figure S7C, http://links.lww.com/HC9/A176). Partial or complete reversion of the antiviral impact of FXR ligands on HBV was also observed following FXR silencing (Figure S7D, http://links.lww.com/HC9/A176). Overall, these results demonstrated that the inhibition of HDV markers induced by FXR ligands was dependent on the presence of FXR, its specific target.

### FXR ligands strongly decrease the secretion of HDV particles in dHepaRG cells and PHH coinfected with HBV and HDV

**FIGURE 4:**
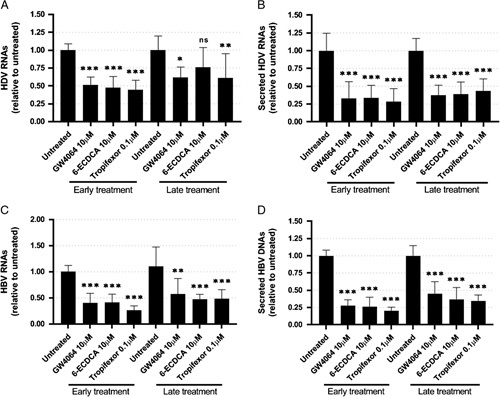
Farnesoid X receptor alpha ligands inhibit secretion of HDV RNA in differentiated HepaRG cells coinfected with HBV and HDV. dHepaRG cells were coinfected with HBV and HDV using 100 and 10 viral genome equivalent per cell, respectively. Cells were treated with 10 µM of GW4064 or 6-ECDCA or 0.1 µM of tropifexor, 1 day postinfection for early treatment and 5 days postinfection for late treatment. Cells and supernatants were harvested 10 days after treatment. Total cellular RNAs and viral nucleic acids in supernatants were extracted. Intracellular (A) and secreted (B) HDV RNAs were quantified by RT-qPCR. Intracellular HBV RNAs (C) and secreted HBV DNA (D) were also quantified by RT-qPCR and qPCR, respectively. Results are the mean±SD of 3 experiments each performed with 3 biological replicates. Data are normalized to the untreated conditions for early and late treatments. Student *t* test, *p<0.05, **p<0.01, ***p<0.001. Abbreviation: ns, not significant.

Next, we wanted to evaluate the effect of FXR ligands on HDV particle secretion corresponding to the late step of the HDV life cycle. To this end, HBV/HDV coinfections were performed in dHepaRG cells. Following infection, cells were treated at day 1 (early treatment) or at day 5 postinfection (late treatment). Following a 10-day treatment, cells and supernatants were collected for further analyses. As previously observed in HDV-monoinfected cells, all 3 FXR ligands decreased by around 50% the amount of intracellular HDV RNAs following early treatment, whereas after late treatment, this decrease was lower or not significant (Figure 4A). Analysis of secreted viral parameters showed that a decrease of secreted HDV RNAs in supernatants was observed following early treatment with FXR ligands by around 60%. In contrast to intracellular HDV RNAs, secreted HDV RNAs were reduced following late treatment in the same proportions as early treatment (Figure 4B). A decrease in total HBV RNAs and secreted HBV DNAs was reported by more than 50% after early and late treatments with FXR agonists (Figure 4C, D). As expected, a decrease of FXR mRNA and an induction of BSEP mRNA were observed following treatment with FXR agonists (Figure S8, http://links.lww.com/HC9/A176). Overall, these results showed that treatment with FXR ligands affected the secretion step of the HDV life cycle, even when treatment was performed late at the peak of HDV replication.

### FXR ligand GW4064 decreases specific infectivity of HDV particles

**FIGURE 5:**
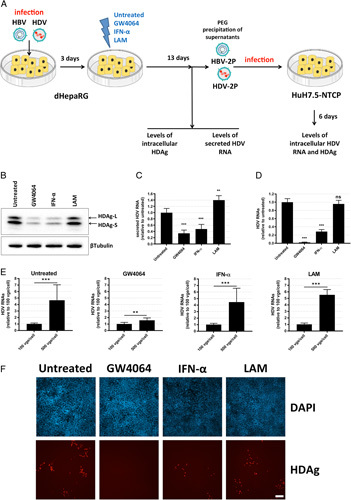
GW4064 reduces the infectivity of HDV particles. Differentiated HepaRG cells were coinfected with HBV and HDV with 500 viral genome equivalent (vge) per cell for HBV and 50 vge per cell for HDV. Cells were treated or not 3 days later with GW4064 (10 µM), IFN-α (500 IU/mL), or lamivudine (LAM, 10 µM) for 10 days. (A) Schematic representation of the experimental procedure. (B) Differentiated HepaRG (dHepaRG) cells were collected and the level of intracellular HDAg was analyzed by the western blot. (C) Supernatants of infected dHepaRG cells were collected, concentrated by PEG precipitation, and the levels of extracellular HDV RNAs (called HDV-2P for second passage) were assessed by qRT-PCR analyses. (D–F) Naive HuH7.5-NTCP cells were infected with the different concentrated supernatants (HDV-2P) with (D) 500 vge cell or (E) the indicated vge per cell. Six days later, (D, E) levels of intracellular HDV RNAs were assessed by RT-qPCR analyses; (F) cells were stained with DAPI and anti-HDAg antibodies. Scale bar: 200 µm. Results of RT-qPCR are the mean±SD of 3 independent experiments each performed with 3 biological replicates. Student *t* test, **p<0.01, ***p<0.001. Abbreviations: IFN, interferon; ns, not significant; NTCP, Na+-taurocholate cotransporting polypeptide.

Next, we wanted to determine the impact of FXR ligands on specific infectivity of secreted HDV particles. This was particularly important to investigate as the continuous infection of naive or already infected hepatocytes by HBs-bearing infectious HDV virions is thought to play a major role in HDV persistence in patients, as opposed to HBV for which persistence is a less dynamic process associated with long-lived cccDNA.^30^ To this end, HBV/HDV coinfection experiments were performed in dHepaRG cells according to the protocol depicted in (Figure 5A). Cells were treated for 10 days with either GW4064, IFN-α, or a specific HBV-polymerase inhibitor (lamivudine). At the end of treatment, cells and supernatants of dHepaRG cells were collected for further analysis. First, western blot analysis of the levels of intracellular HDV antigens was performed to confirm the antiviral effect of FXR ligands. As previously observed, GW4064 and IFN-α treatment strongly decreased the amount of intracellular HDAg-S and HDAg-L, while lamivudine had no effect, as expected (Figure 5B). Quantification of HDV RNA in supernatants by RT-qPCR revealed that the secretion of HDV genomes decreased by 65% following GW4064 treatment, and 52% with IFN-α treatment (Figure 5C). Of note, lamivudine slightly and unexpectedly stimulated HDV viral secretion through a mechanism that still needs to be clarified.

To determine the specific infectivity of secreted HDV, viral particles in supernatants were concentrated using PEG precipitation, and naive HuH7.5-NTCP cells were infected with the same viral genome equivalents for each condition. Six days postinfection, intracellular HDV RNA was quantified by RT-qPCR, and HDV antigen production was evaluated by IF staining. Total intracellular HDV RNAs in HuH7.5-NTCP cells decreased by 98% and 71% when the cells were infected with supernatants from infected dHepaRG cells treated with GW4064 and IFN-α, respectively (Figure 5D). By contrast, treatment with lamivudine did not affect specific infectivity of HDV particles. Interestingly, when infecting naive HuH7.5-NTCP cells with a different inoculum (ie, 100 and 500 viral genome equivalent per cell), a dose response was observed with supernatants from dHepaRG cells treated with either IFN-α, lamivudine, or vehicle, whereas this was not the case for GW4064 (Figure 5E). Finally, IF staining confirmed the RT-qPCR data as HDAg-positive cells could hardly be detected among HuH7.5-NTCP cells infected with supernatants from dHepaRG cells treated with GW4064 (Figure 5F).

Supernatants were also subjected to isopycnic centrifugation on iodixanol density gradients. Fractions collected were tested for density, HDV RNA, and HBV DNA by qPCR, HBsAg by ELISA, and HDAg and HBsAg by western blot. No modification in the distribution and densities of fractions containing HDV RNA, HBV DNA, HDAg, and HBsAg was observed following treatment with GW4064, IFN-α, and lamivudine compared with untreated cells (Figure S9, http://links.lww.com/HC9/A176). Similar experiments were performed with FXR ligands 6-ECDCA and tropifexor to confirm the results obtained with GW4064. Following infection of naive HuH7.5-NTCP cells with viruses produced in dHepaRG cells treated with 6-ECDCA and tropifexor, a decrease of intracellular HDV RNAs by more than 95% was also observed, indicating that the specific infectivity of secreted HDV particles was affected by all 3 FXR ligands used in this study (Figure S10A, B, http://links.lww.com/HC9/A176). A comparable decrease of infectivity was observed when naïve dHepaRG cells were infected with HDV viruses produced in the same conditions of treatment (Figure S10C, http://links.lww.com/HC9/A176). Altogether, these results showed that FXR ligands not only decreased secretion of HDV particles in infected dHepaRG cells but also impaired their specific infectivity.

## DISCUSSION

This study shows that in the most relevant *in vitro* models of HDV infection, treatment with FXR ligands moderately reduces early steps of HDV intracellular replication, more efficiently inhibits secretion of HDV virions, and drastically inhibits the specific infectivity of secreted viral particles. Target engagement was confirmed by the use of several ligands with different chemical structures and FXR loss of function experiments.

In HDV monoinfected dHepaRG cells, the amount of genomic and antigenomic HDV RNA, which are key markers of HDV replication, was reduced by the treatment with FXR ligands. This demonstrates that, at least for the HDV genome replication steps, the inhibitory effect of FXR ligands is unrelated to their anti-HBV activity, which has been previously described.^20^,^21^ In HBV/HDV coinfection models, it is shown that FXR ligands induced a decrease in HDV RNA secretion. This impaired secretion of hepatitis delta virions may result from the decrease of all markers of HDV replication (HDV RNAs and proteins), combined with the inhibition of HBs synthesis, not excluding other mechanisms that remain to be identified.

One major impact of FXR activation is the pronounced decrease in specific infectivity (by more than 95%) of virions produced by coinfected dHepaRG cells. HDV RNA-containing particles were indeed poorly efficient to infect and/or initiate viral replication in naive HuH7.5-NTCP cells, suggesting key modifications of virion composition following treatment. No shift in the density of RNA-, HDAg-, or HBs-containing fractions secreted upon FXR engagement could be observed, but a detailed analysis of virion composition and structure is warranted (ie, proteomic, lipidomic, atomic-force microscopy). Importantly, this reduction of specific infectivity obtained with GW4064, 6-ECDCA, and tropifexor was superior to that obtained with the SoC IFN-α.

One limitation of the study is that all experiments were performed using a unique HDV strain of genotype 1 with a particular history.^31^ This strain is commonly used in HDV research; however, it would be of great interest to study the antiviral effect of FXR ligands on other HDV genotypes.

FXR belongs to the NR superfamily, a group of transcription factors sharing a common modular structure that includes a DNA-binding domain and a ligand-binding domain. Binding of ligands on FXR may result in FXR activation and modulation of FXR target gene expression, in modifications of FXR conformation leading to either promotion or destabilization of interactions with nucleic acids or protein partners and finally in repression of FXR expression through a negative feedback loop. HDV RNAs, HDAg-S, and host components associate to form HDV viral replication/transcription complexes. In particular, it was shown that host DNA-dependent RNA polymerase II could be recruited onto HDV RNA genome and drive transcription, thus indicating that HDV can hijack host factors known to interact with dsDNA and not dsRNA.^32^ To our knowledge, FXR activation does not directly modulate the expression of DNA-dependent RNA polymerase II, but it is well established that ligand binding induces a conformational change of NRs, leading to the recruitment of various coactivators, some of them with acetylase and methylase activities. Histone acetylation and methylation status is then modified by these coactivators with consequences on the transcription of target genes. Whether this could apply to the regulation of HDV transcription remains to be explored, as the regulation of RNA polymerase II on the HDV genome is far from being fully understood. However, one can speculate that FXR may interact or interfere with viral or cellular components within HDV replication complexes, and that this interaction may be modified by FXR ligands.

Alternatively, FXR activation by ligands may specifically modulate the expression of genes involved in pathways playing a key role in the HDV life cycle. Such modulations may lead to altered replication and transcription of the HDV genome and/or modifications of the structure and composition of secreted particles during the virion assembly. FXR is an essential regulator of several liver metabolic pathways, its role in regulating lipid metabolism (in particular BA and lipoprotein metabolism) and glucose metabolism being extensively studied. However, its role is not limited to these pathways, as FXR has also been linked to the regulation of liver regeneration and innate immunity.^17^,^33^ Cellular pathways essential for the HDV life cycle are still poorly known to date. Recently, a screening based on RNA interference identified several cellular pathways involved in HDV replication such as HIF-1 signaling pathway, pyrimidine biosynthesis, insulin resistance, or glycosaminoglycan biosynthesis.^34^ Protein prenylation pathway is also known to play a key role during HDV virion assembly as farnesylation of HDAg-L by cellular farnesyl-transferases favors HDAg-L localization to the ER and association with HBsAg. Interestingly, some links exist between prenylation and BA pathways. Besides geranylgeranyl, farnesyl can also be transformed into squalene, an essential biochemical precursor for steroids (including cholesterol and BA) or farnesol. Farnesol was the first ligand identified for FXR^35^ but, following the identification of BA as new FXR ligands, studies on relationships between FXR and prenylation pathway were given less priority. Given the major impact of FXR ligands on HDV virion infectivity, studying the impact of FXR activation on HDAg-L may be of great interest. Moreover, overexpression of HDAg-L has also been shown to inhibit HDV viral replication,^11^ and FXR-induced putative modulations of HDAg-L activity might also interfere with the replication step of the HDV cycle

In conclusion, our results revealed that FXR agonists inhibit early steps of intracellular HDV genome replication in *in vitro* infected dHepaRG and PHH, with an antiviral activity comparable to that of IFN-α. In HBV/HDV coinfected cells, FXR ligation decreases HDV secretion and even more significantly reduces the specific infectivity of secreted HDV virions, leading altogether to an antiviral phenotype far superior to that obtained with IFN-α, the current SoC in HDV patients. After HBV, HDV is the second hepatotropic virus that seems to be very sensitive to therapeutic FXR agonization, which suggests that this NR may be broadly involved in host antiviral responses. The majority of studies focus on FXR metabolic functions, and a more detailed analysis of mechanisms underlying FXR antiviral activity has to be pursued.

To date, the therapeutic options for patients infected with HDV are limited to Peg-IFN-α and bulevirtide, an entry inhibitor. Other molecules are currently under evaluation: Peg-IFN-λ1, lonafarnib, a prenylation inhibitor that impairs HDV secretion, and REP 2139, a nucleic acid polymer that inhibits HBs, HBV, and HDV secretion. Combined therapies will likely be necessary to achieve an SVR in the majority of patients. As constant reinfection of hepatocytes is thought to play a major role in HDV persistence, the strong inhibition of virion production and spreading following treatment with FXR ligands opens promising therapeutic perspectives for these molecules in the treatment of hepatitis delta. FXR is an attractive target for the development of antiviral molecules as some FXR ligands have already been approved for patients with primary biliary cholangitis or are currently in clinical evaluation in patients with NASH.^36^ Moreover, one FXR ligand is currently in a phase II clinical trial in patients with chronic HBV infection (Clinical Trial identifiers NCT04365933 and NCT04465916). In this context, the clinical evaluation of FXR modulators in HBV/HDV coinfected patients, alone or in combination, could be a successful strategy.

## Supplementary Material

SUPPLEMENTARY MATERIAL
