# Supplementary material for: Farnesoid X receptor alpha ligands inhibit HDV in vitro replication and virion infectivity
Source: Hepatol Commun. 2023 Apr 14;7(5):e0078. doi: 10.1097/HC9.0000000000000078 (PMC10109841; doi:10.1097/HC9.0000000000000078)
Supplement: SUPPLEMENTARY MATERIAL [file hc9-7-e0078-s002.pdf]

Supplementary figures

Figure S1

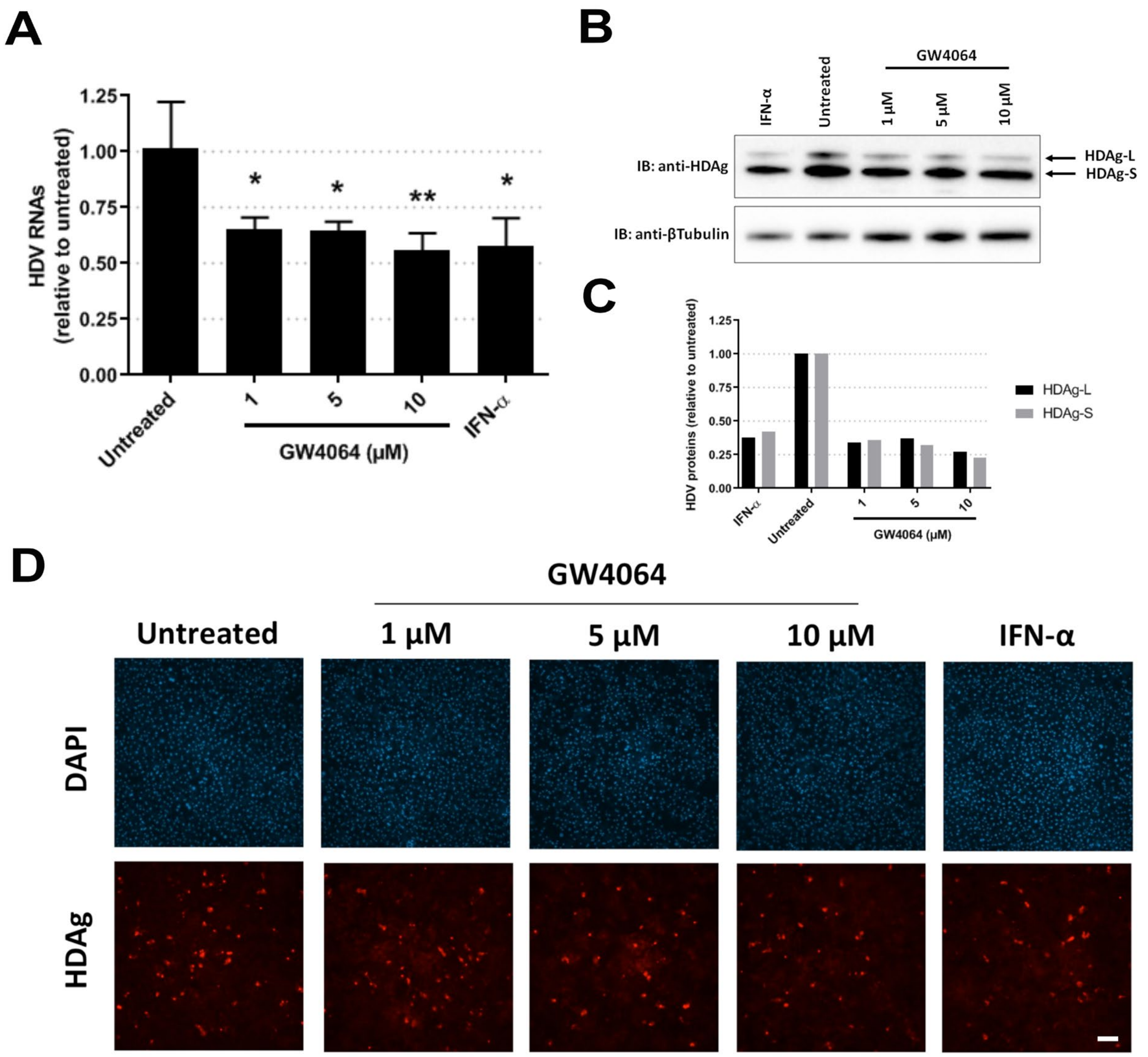

Figure S2

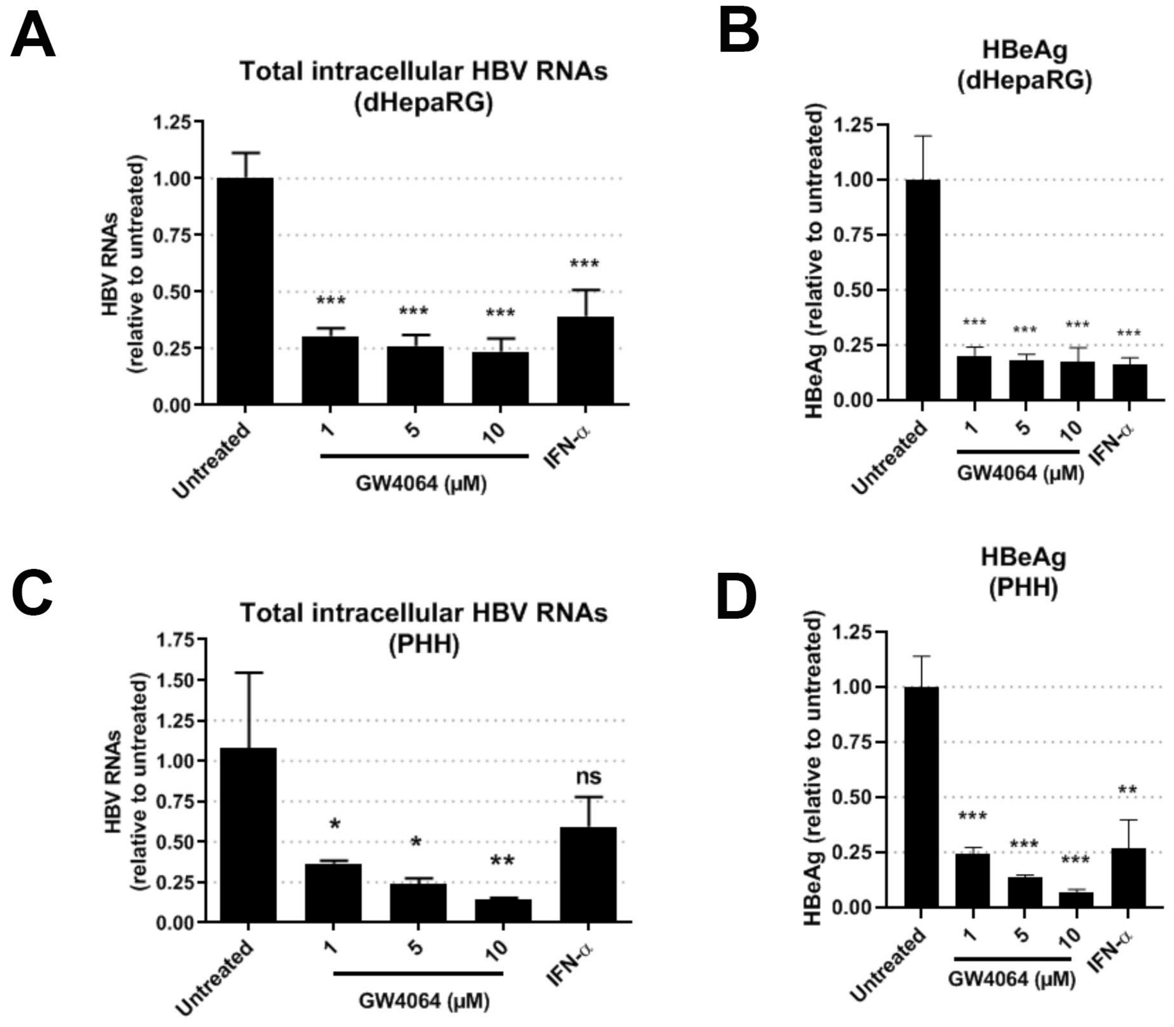
A
Total intracellular HBV RNAs
(dHepaRG)
HBV RNAs
(relative to untreated)
1.25
1.00
0.75
0.50
0.25
0.00
***
***
***
***
Untreated
1
5
10
IFN-α
GW4064 (µM)
B
HBeAg
(dHepaRG)
HBeAg (relative to untreated)
1.25
1.00
0.75
0.50
0.25
0.00
***
***
***
***
Untreated
1
5
10
IFN-α
GW4064 (µM)
C
Total intracellular HBV RNAs
(PHH)
HBV RNAs
(relative to untreated)
1.75
1.50
1.25
1.00
0.75
0.50
0.25
0.00
*
*
**
ns
Untreated
1
5
10
IFN-α
GW4064 (µM)
D
HBeAg
(PHH)
HBeAg (relative to untreated)
1.25
1.00
0.75
0.50
0.25
0.00
***
***
***
**
Untreated
1
5
10
IFN-α
GW4064 (µM)

Figure S3

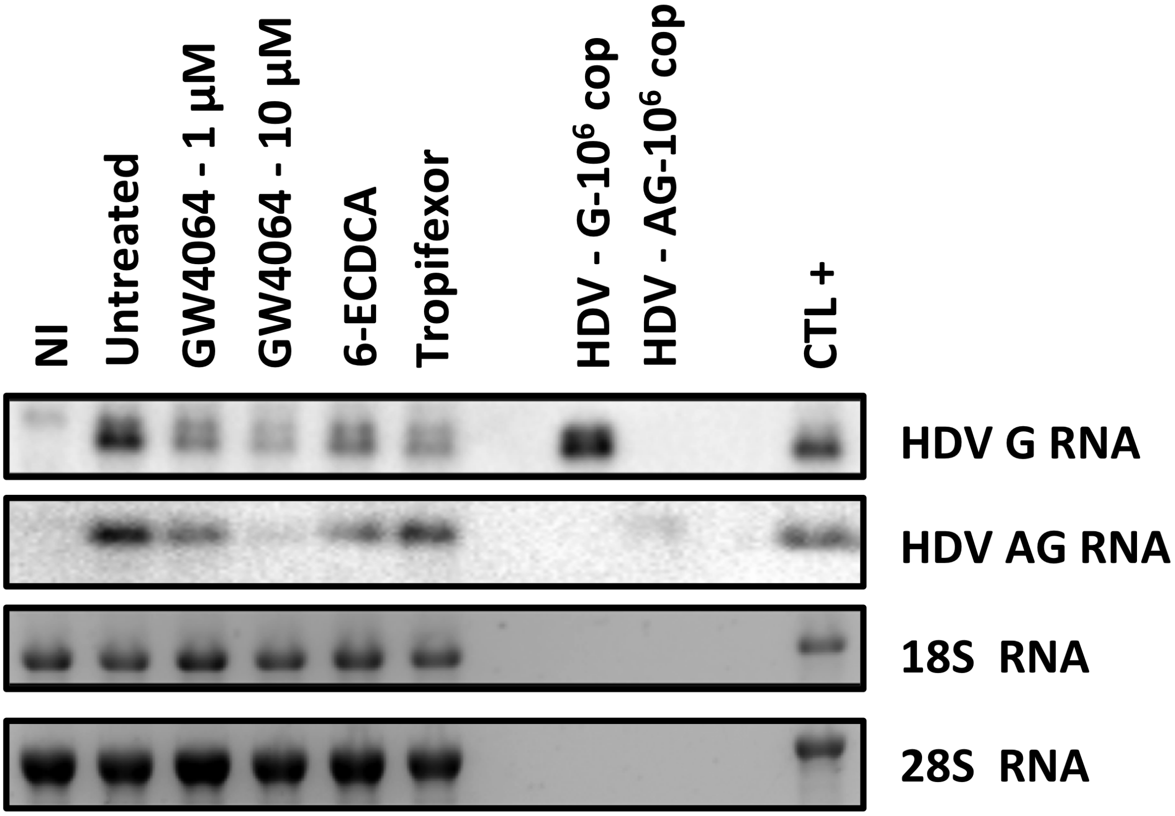
NI
Untreated
GW4064 - 1 µM
GW4064 - 10 µM
6-ECDCA
Tropifexor
HDV - G-10⁶ cop
HDV - AG-10⁶ cop
CTL +
HDV G RNA
HDV AG RNA
18S RNA
28S RNA

Figure S4

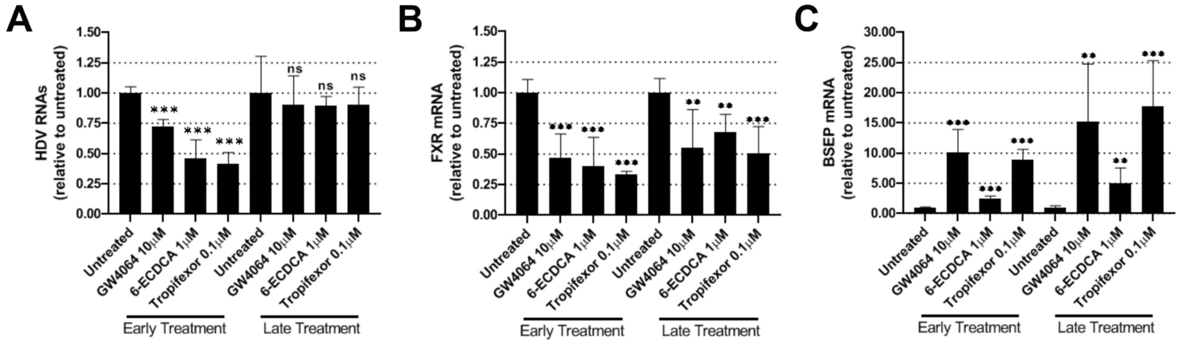
A
HDV RNAs
(relative to untreated)
B
FXR mRNA
(relative to untreated)
C
BSEP mRNA
(relative to untreated)
Untreated
GW4064 10μM
6-ECDCA 1μM
Tropifexor 0.1μM
Early Treatment
Late Treatment
ns

Figure S5

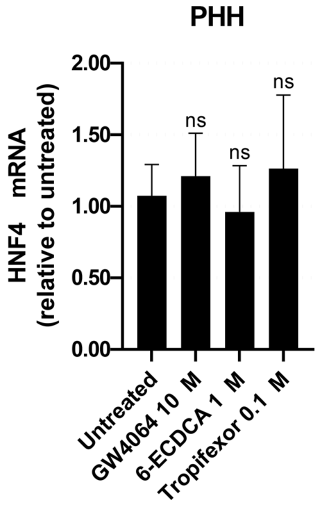
PHH
HNF4 mRNA
(relative to untreated)
2.00
1.50
1.00
0.50
0.00
ns
ns
ns
Untreated
GW4064 10 M
6-ECDCA 1 M
Tropifexor 0.1 M

Figure S6

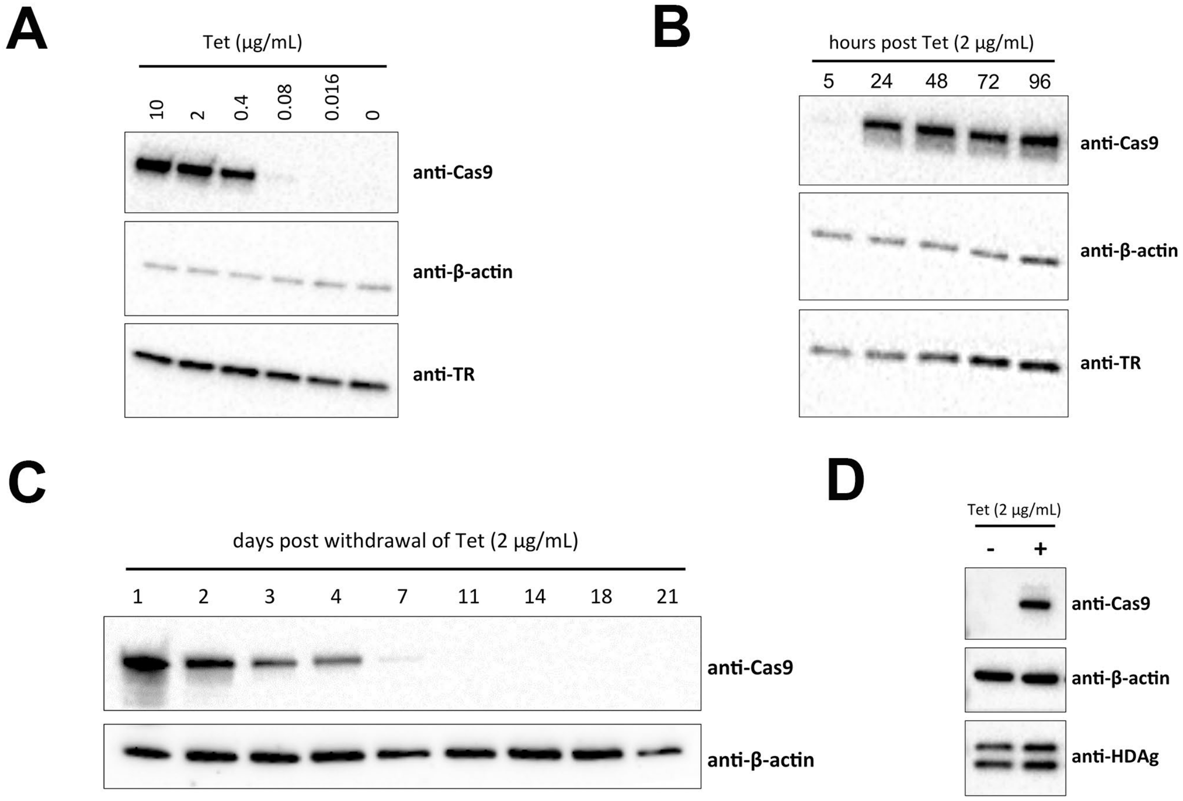
A
Tet (µg/mL)
10
2
0.4
0.08
0.016
0
anti-Cas9
anti-β-actin
anti-TR
B
hours post Tet (2 µg/mL)
5
24
48
72
96
anti-Cas9
anti-β-actin
anti-TR
C
days post withdrawal of Tet (2 µg/mL)
1
2
3
4
7
11
14
18
21
anti-Cas9
anti-β-actin
D
Tet (2 µg/mL)
-
+
anti-Cas9
anti-β-actin
anti-HDAg

Figure S7

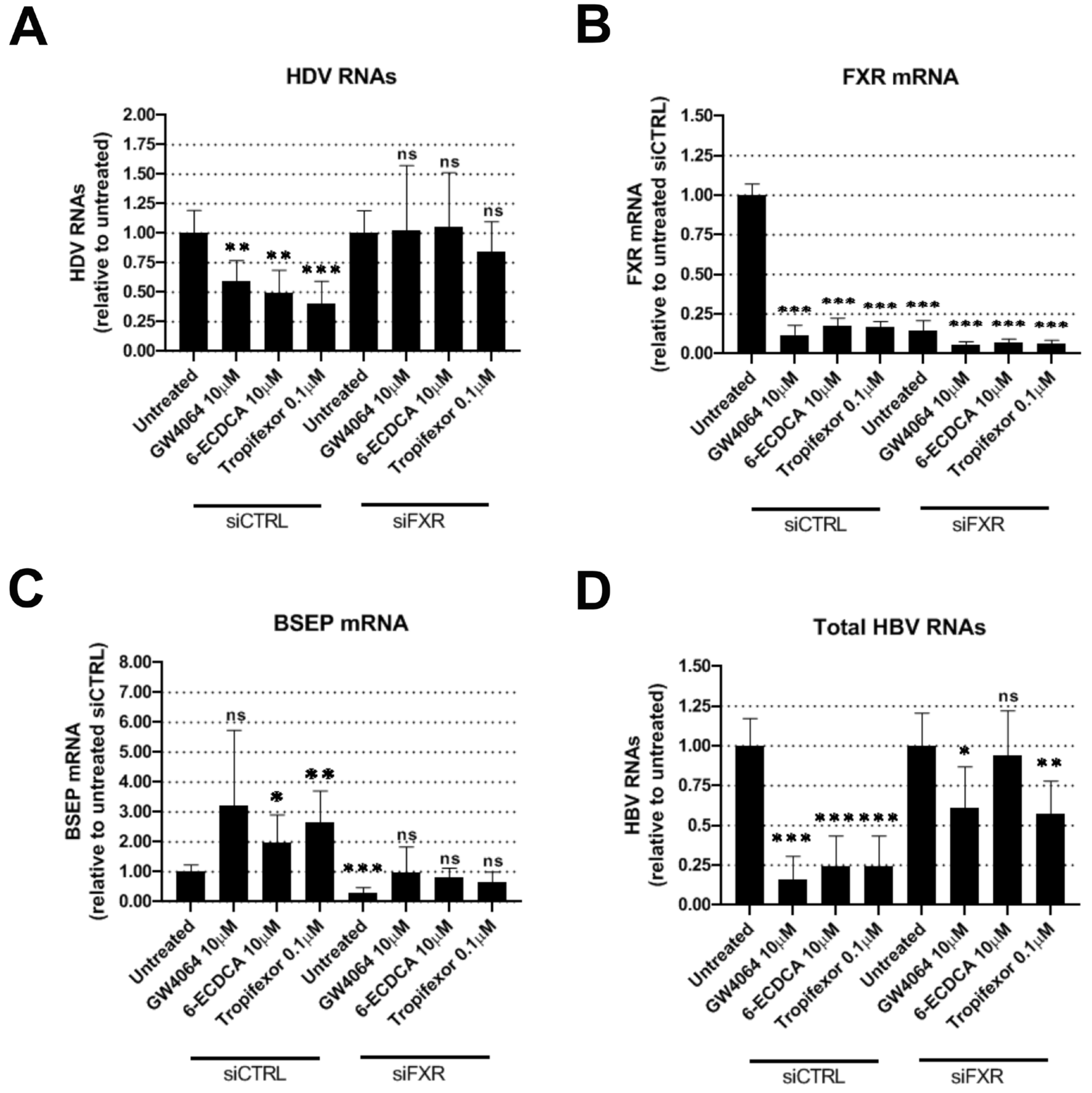
A
HDV RNAs
HDV RNAs
(relative to untreated)
B
FXR mRNA
FXR mRNA
(relative to untreated siCTRL)
C
BSEP mRNA
BSEP mRNA
(relative to untreated siCTRL)
D
Total HBV RNAs
HBV RNAs
(relative to untreated)
Untreated
GW4064 10μM
6-ECDCA 10μM
Tropifexor 0.1μM
siCTRL
siFXR
ns

Figure S8

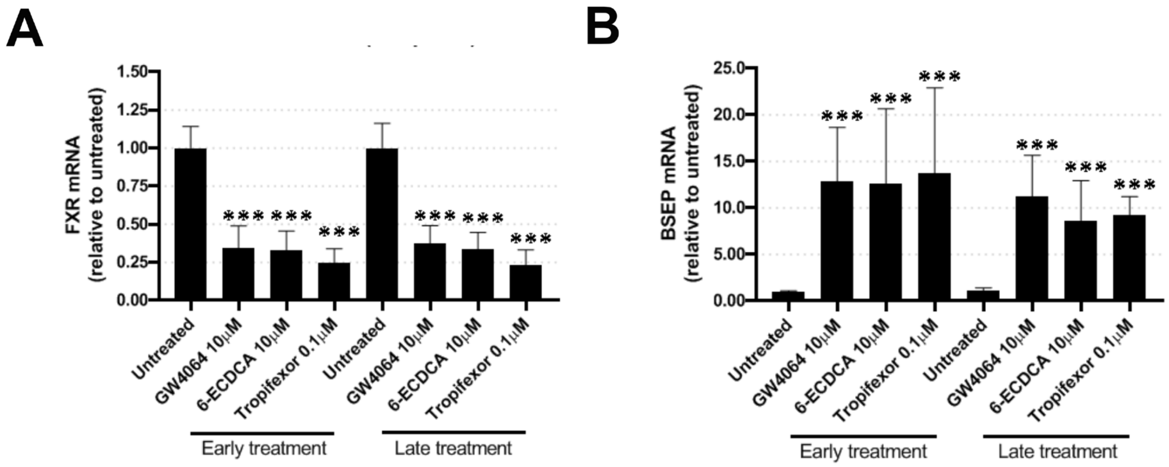
A
FXR mRNA
(relative to untreated)
1.50
1.25
1.00
0.75
0.50
0.25
0.00
***
Untreated
GW4064 10μM
6-ECDCA 10μM
Tropifexor 0.1μM
Early treatment
Late treatment
B
BSEP mRNA
(relative to untreated)
25.0
20.0
15.0
10.0
5.00
0.00

Figure S9.

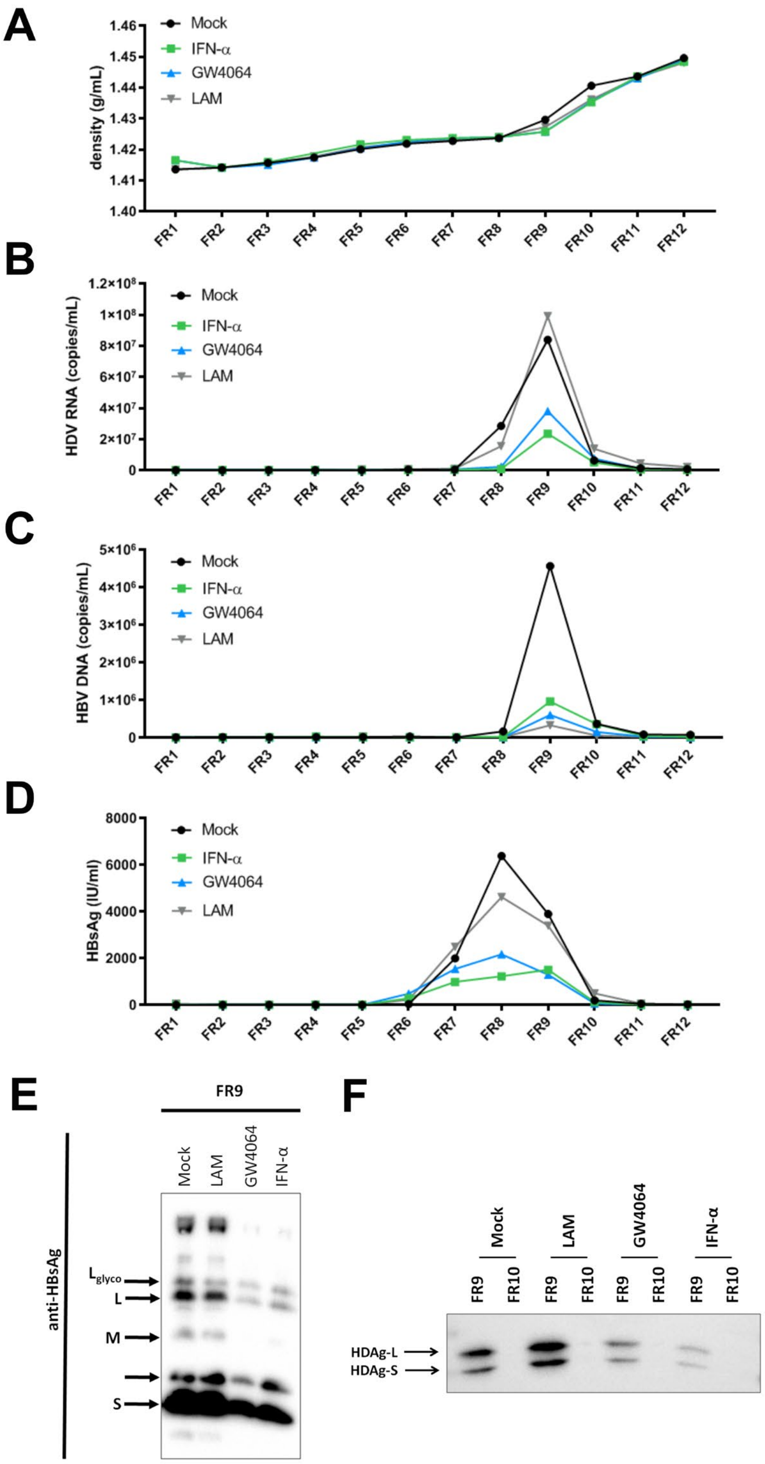
A
Mock
IFN-α
GW4064
LAM
density (g/mL)
FR1 FR2 FR3 FR4 FR5 FR6 FR7 FR8 FR9 FR10 FR11 FR12
B
HDV RNA (copies/mL)
C
HBV DNA (copies/mL)
D
HBsAg (IU/ml)
E
FR9
Mock LAM GW4064 IFN-α
anti-HBsAg
L_{glyco}
L
M
S
F
HDAg-L
HDAg-S

Figure S10.

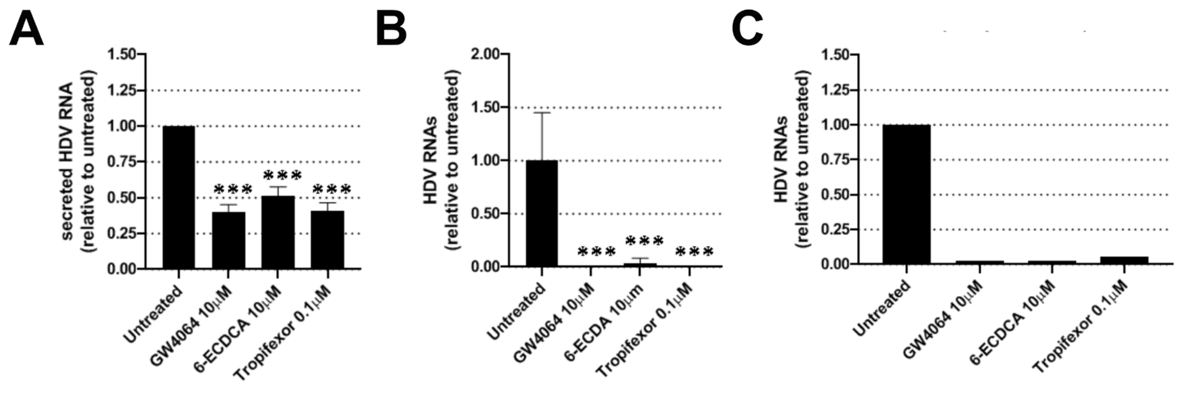
A
secreted HDV RNA (relative to untreated)
1.50
1.25
1.00
0.75
0.50
0.25
0.00
***
***
***
Untreated
GW4064 10μM
6-ECDCA 10μM
Tropifexor 0.1μM
B
HDV RNAs (relative to untreated)
2.00
1.50
1.00
0.50
0.00
***
***
***
Untreated
GW4064 10μM
6-ECDA 10μm
Tropifexor 0.1μM
C
HDV RNAs (relative to untreated)
1.50
1.25
1.00
0.75
0.50
0.25
0.00
Untreated
GW4064 10μM
6-ECDCA 10μM
Tropifexor 0.1μM
