# Supplementary material for: Farnesoid X receptor alpha ligands inhibit HDV in vitro replication and virion infectivity
Source: Hepatol Commun. 2023 Apr 14;7(5):e0078. doi: 10.1097/HC9.0000000000000078 (PMC10109841; doi:10.1097/HC9.0000000000000078)
Supplement: SUPPLEMENTARY MATERIAL [file hc9-7-e0078-s001.docx]

**Supplementary methods**

***Nucleic acid quantification***

Total intracellular RNAs were extracted by using the NucleoSpin RNA II kit according to the manufacturer’s instructions (Macherey-Nagel). DNAs and RNAs from HBV or HDV particles were isolated from cell supernatants by using the NucleoSpin RNA Virus kit (Macherey-Nagel) according to the manufacturer's instructions. RNA was reverse-transcribed using either High-Capacity RNA-to-cDNA kit (ThermoFisher Scientific) or Maxima RT (Life Technologies). Quantitative PCR for HDV and HBV were performed as previously described (1,2). FXR and BSEP mRNAs were quantified by qPCR as previously described (3). HNF4A mRNA was quantified by using the following primers: 5’-CGTGGAGGCAGGGAGAATGCGA-3’ and 5’-TTCTGATGGGGACGTGTCATTGC-3’. Statistical analyses were performed using Student’s t-test: *p < 0.05, **p < 0.01, ***p < 0.001, ns: not significant.

Northern blot for detection of HDV genomic and antigenomic RNAs was essentially performed as previously described (2). Briefly, purified RNA was denatured at 50°C for one hour with glyoxal (Life Technologies), subjected to electrophoresis through a phosphate 1.2% agarose gel and transferred to a nylon membrane (Amersham Nþ, GE). Membrane-bound RNA was hybridized to digoxigenin(DIG)-labeled HDV-specific probes. Quantitative analysis of HDV RNA was achieved by phosphorimager scanning (Typhoon Fla 9500, GE); 18S and 28S rRNA detection was used as loading control.

***ELISA***

Commercial immunoassay kits (Autobio Diagnostics Co., China) were used for HBsAg and HBeAg quantification in cell culture supernatants. Results are presented as a ratio to a control sample, described for each experiment. Cut-offs for these ELISA were 0.05 IU/mL for HBsAg and 0.1 PEIU/mL for HBeAg.

***Western blotting***

For the detection of FXR protein by western blot, cells were lysed by using buffer containing 10 mM HEPES, 10mM NaOH (pH 7.5), 10 mM KCl, 1.5 mM MgCl2, 0.3% NP40, protease inhibitor mixture cocktail (P8340-Merck) and Pierce™ universal nuclease for cell lysis (#88701, Thermo Fisher Scientific). Samples were then diluted with 4X Laemmli sample buffer (Biorad, #1610747) and supplemented with 1mM dithiothreitol. Mouse anti-FXR (clone A9033A, ThermoFisher Scientific) and anti-GAPDH (clone 1E6D9, Proteintech) antibodies were used, followed by horseradish peroxidase (HRP) detection with SuperSignal™ West Pico or Femto chemiluminescent substrate (Thermo Fisher Scientific) according to manufacturer’s instructions. HRP signal detection was determined electronically using the Syngene PXi Image system (Ozyme).

For the detection of HDV antigens, cells were washed with Phosphate buffered saline (PBS) and harvested in RIPA lysis buffer (Tris-HCl pH 7.5 10mM, NaCl 140mM, EDTA 1mM, EGTA 0.5mM, 1% Triton X100, 0.1% SDS, 0.1% Na-Deoxycholate) containing protease inhibitors (Protein Cocktail Inhibitors from Sigma-Aldrich). Clarified lysates were subjected to SDS-PAGE and Western Blot transfer onto nitrocellulose membranes using the iBlot2 apparatus according to the manufacturer (Thermofisher Scientific). The polyclonal anti-HDAg serum, obtained by rabbit immunization (with a proprietary strategy), was a kind gift from Janssen. Anti-human βTubulin was purchased from Abcam and used as a loading control. Detection was performed with Gel Doc XR þ System (BioRad) and images were analyzed with ImageJ software.

Anti-HBsAg (H166), used for HBs detection in gradient fractions, has been previously described (4).

The references of antibodies used in this study are provided in Supplementary Table 1.

***Immunofluorescence***

Immunofluorescence (IF) experiments were performed as previously described (2). Cells were fixated with formaldehyde 4% and permeabilized by Triton 0.1%. Nuclei were stained with 4.6-diamidino-2-phenylindole (DAPI). The anti-HDAg antibody used was the same as for western blots. Secondary antibody used was goat anti-human Alexa Fluor 555 (Invitrogen). Images were obtained by epifluorescence microscopy (Nikon eclipse TE2000-E; Nikon).

***siRNA transfections***

siRNA transfections into dHepaRG cells were performed using Lipofectamine RNAiMAX (Thermofisher Scientific) according to manufactor’s guidelines. siCTRL (ON-TARGETplus Non-targeting pool) and siFXR (ON-TARGETplus Human NR1H4 siRNA SMART pool) were purchased from Dharmacon (Horizon Discovery, Cambridge, UK).

**Supplementary references**

1. Lucifora J, Xia Y, Reisinger F, Zhang K, Stadler D, Cheng X, et al. Specific and nonhepatotoxic degradation of nuclear hepatitis B virus cccDNA. Science. 2014;343:1221–1228.

2. Alfaiate D, Lucifora J, Abeywickrama-Samarakoon N, Michelet M, Testoni B, Cortay J-C, et al. HDV RNA replication is associated with HBV repression and interferon-stimulated genes induction in super-infected hepatocytes. Antiviral Res. 2016;136:19–31.

3. Mouzannar K, Fusil F, Lacombe B, Ollivier A, Ménard C, Lotteau V, et al. Farnesoid X receptor-α is a proviral host factor for hepatitis B virus that is inhibited by ligands in vitro and in vivo. FASEB J. Off. Publ. Fed. Am. Soc. Exp. Biol. 2019;33:2472–2483.

4. Chen YC, Delbrook K, Dealwis C, Mimms L, Mushahwar IK, Mandecki W. Discontinuous epitopes of hepatitis B surface antigen derived from a filamentous phage peptide library. Proc. Natl. Acad. Sci. U. S. A. 1996;93:1997–2001.

Figure S1. FXR ligand GW4064 decreases the levels of intracellular HDV RNAs and proteins in HBV-infected PHH superinfected with HDV. PHH were infected with HBV at a MOI of 100 vge per cell and 4 days later with HDV at a MOI of 10 vge per cell. 3 days post-HDV infection, cells were treated with 1, 5 or 10 µM of GW4064, IFN-α (1000 IU/mL) or not. Cells were harvested 10 days post-treatment for cellular RNA and protein extraction or fixed with formaldehylde for immunofluorescence analyses. Intracellular HDV RNAs were quantified (A). Results are the mean +/- SD of one experiment performed with three biological replicates. Student’s t-test, *p < 0.05, **p < 0.01, ***p < 0.001. Analysis of the levels of HDAg by western blot analyses were performed using anti-HDAg antibodies and anti-B-tubulin antibodies as a loading control (B). Densitometry analyses are presented as ratios of HDAgs normalized to the levels of B-tubulin (C). Immunofluorescence analyses were performed using anti-HDAg antibodies and nuclei DAPI staining (D). Scale bar: 200µm.

Figure S2. FXR ligand GW4064 decreases the levels of HBV replication markers in HBV-infected dHepaRG and PHH superinfected with HDV. dHepaRG cells or PHH were infected and treated as described for Fig. 1 and S1. Cells and supernatants were harvested 10 days post-treatment for quantification of intracellular HBV RNAs (A: dHepaRG cells ; C: PHH) and secreted HBe antigens (B: dHepaRG cells ; D: PHH). Results are the mean +/- SD of three experiments (dHepaRG) and one experiment (PHH) each performed with three biological replicates. Student’s t-test *p < 0.05, **p < 0.01, ***p < 0.001, ns: not significant.

**Figure S3.** **FXR ligands decrease the levels of genomic and antigenomic HDV RNAs in HDV-monoinfected dHepaRG cells.** dHepaRG cells were infected with HDV at a MOI of 25 vge per cell. From day 4 to day 11 post-infection, cells were treated with 10 µM of GW4064, 10 µM of 6-ECDCA and 1 µM of tropifexor. Cells were harvested at day 11 post HDV infection and total RNAs were extracted. HDV genomic and antigenomic RNAs were analyzed by Northern Blot. NI: non infected; G RNA: genomic RNA; AG RNA: antigenomic RNA.

Figure S4. FXR ligands inhibit early phases of HDV infection in HDV-monoinfected PHH. PHH were infected with HDV at a MOI of 10 vge per cell. Cells were treated with 10µM of GW4064, 1µM of 6-ECDCA or 0.1µM of tropifexor, either 1 day post-infection (early treatment) or 5 days post-infection (late treatment). Cells were harvested 10 days post-treatment. Total cellular RNAs were extracted and intracellular HDV RNAs (A), FXR mRNA (B) and BSEP mRNA (C) were quantified by RT-qPCR. Results are the mean +/- SD of two experiments each performed with 3 biological replicates. Data are normalized to untreated conditions for early and late treatments. Student’s t-test , *p<0.05, **p<0.01, ***p<0.001, ns: not significant.

Figure S5. FXR ligands do not modify HNF4A mRNA expression in PHH. PHH were treated for 10 days with GW4064 at 10µM, 6-ECDCA at 1µM and tropifexor at 0.1 µM. Total cellular RNAs were extracted and the level of HNF4A mRNA was quantified by RT-qPCR. Results are the mean +/- SD of three independent experiments each performed with three biological replicates. Student’s t-test, ns: not significant.

Figure S6. Characterization of HepaRG-TR-Cas9 cells. (A) dHepaRG-TR-Cas9 cells were treated with the indicated concentrations of Tetracyclin (Tet) for 3 days. (B) dHepaRG-TR-Cas9 cells were treated with Tet and collected at the indicated time. (C) dHepaRG-TR-Cas9 cells were treated with Tet for 24h and cells were collected at the indicated time after withdrawal of Tet. (D) dHepaRG-TR-Cas9 cells were treated or not with Tet for 1 day before infection with HDV at 100 vge/cell. Cells were collected at day 6 post-infection. (A, B, C, D) Western blot analyses were performed using the indicated antibodies.

Figure S7. FXR silencing by siRNA abolishes the antiviral effect of FXR ligands on intracellular HDV RNAs in dHepaRG cells. dHepaRG cells were coinfected with HBV at a MOI of 100 vge per cell and with HDV at a MOI of 10 vge per cell. 1 day post-infection, cells were transfected with a non-targeting siRNA control (siCTRL) or siRNA targeting FXR expression (siFXR). Following siRNA transfection, cells were treated or not for 7 days with GW4064, 6-ECDCA or tropifexor. Cells were collected and intracellular RNAs were extracted. The levels of HDV RNAs (A), FXR mRNA (B), BSEP mRNA (C) and total HBV RNAs (D) were assessed by RT-qPCR analyses. Results are the mean +/- SD of two experiments each performed with 3 biological replicates. Data are normalized to untreated cells transfected with siCTRL. Student’s t-test , *p<0.05, **p<0.01, ***p<0.001, ns: not significant.

Figure S8. FXR ligands decrease FXR mRNA expression and induce BSEP mRNA expression in HBV/HDV coinfected dHepaRG cells. dHepaRG cells were monoinfected with HDV at a MOI of 10 vge per cell or coinfected with HBV and HDV at a MOI of 100 and 10 vge per cell, respectively. Cells were treated with 10µM of GW4064 or 6-ECDCA or 0.1µM of tropifexor, on day 1 post-infection for early treatment and on day 5 post-infection for late treatment. Cells were harvested 10 days post-treatment. Total cellular RNAs were extracted and FXR mRNA (A) and BSEP mRNA (B) were quantified by RT-qPCR. (C) Results are the mean +/- SD of three experiments each performed with 3 biological replicates. Data are normalized to the untreated conditions for early and late treatments. Student’s t-test , ***p<0.001.

**Figure S9.** **FXR ligand GW4064 does not modify buoyant density of secreted HDV particles**. dHepaRG cells were coinfected with HBV and HDV with 500 vge/cell for HBV and 50 vge/cell for HDV. Cells were treated or not 3 days later with GW4064 (10 µM), IFN-α (500 IU/mL) or LAM (10 µM) for 10 days. Supernatants were collected at day 13 post-infection. Supernatants were collected, concentrated by PEG precipitation and submitted to iodixanol gradients overnight. Fractions (FR) were collected and the density (A), the levels of HDV RNAs (B), HBV DNA (C) and HBsAg (D) were analysed by RT-qPCR, qPCR and ELISA. (E, F) WB analyses were performed in selected fractions using anti-HBsAg (E) and anti-HDAg (F) antibodies . LAM: lamivudine.

Figure S10. FXR ligands decrease the infectivity of HDV particles. (A-B) dHepaRG were coinfected with HBV and HDV at 100 vge and 10 vge per cell, respectively. Cells were treated at day 3 post-infection with GW4064 (10μM), 6-ECDCA (10μM) or tropifexor (0.1μM). 13 days post-infection, supernatants were collected and concentrated by PEG-precipitation. (A) The level of extracellular HDV RNAs were analyzed by qRT-PCR analyses. (B) Naïve HuH7.5-NTCP cells were infected with the different concentrated supernatants from treated dHepaRG cells with 500 vge per cell. Six days later, the levels of intracellular HDV RNAs were analysed by RT-qPCR. Results of RT-qPCR are the mean +/- SD of three independent experiments each performed with three biological replicates. Student’s t-test ***p< 0.001. (C) Naïve dHepaRG cells were infected with PEG-precipitated HDV viruses collected from supernatants of dHepaRG cells following a 10-day treatment with the indicated FXR ligands. Six days later, the levels of intracellular HDV RNAs were analyzed by RT-qPCR. Results of RT-qPCR are the mean of one experiment performed with three biological replicates.
